# 4D Printing of NiTi Auxetic Structure with Improved Ballistic Performance

**DOI:** 10.3390/mi11080745

**Published:** 2020-07-31

**Authors:** Hany Hassanin, Alessandro Abena, Mahmoud Ahmed Elsayed, Khamis Essa

**Affiliations:** 1 School of Engineering, Canterbury Christ Church University, Canterbury CT1 1QU, UK; 2 University of Birmingham, Birmingham B15 2TT, UK; Alessandro.Abena@bham.ac.uk (A.A.); k.e.a.essa@bham.ac.uk (K.E.); 3 Department of Industrial and Management Engineering, Arab Academy for Science and Technology and Maritime Transport, P.O Box 1029, Abu Qir, Alexandria 21599, Egypt; dr.mahmoudelsayed12@gmail.com

**Keywords:** 4D printing, NiTi, shape memory alloy, super elasticity, auxetic

## Abstract

Auxetic structures have attracted attention in energy absorption applications owing to their improved shear modulus and enhanced resistance to indentation. On the other hand, four-dimensional (4D) printing is an emerging technology that is capable of 3D printing smart materials with additional functionality. This paper introduces the development of a NiTi negative-Poisson’s-ratio structure with superelasticity/shape memory capabilities for improved ballistic applications. An analytical model was initially used to optimize the geometrical parameters of a re-entrant auxetic structure. It was found that the re-entrant auxetic structure with a cell angle of −30° produced the highest Poisson’s ratio of −2.089. The 4D printing process using a powder bed fusion system was used to fabricate the optimized NiTi auxetic structure. The measured negative Poisson’s ratio of the fabricated auxetic structure was found in agreement with both the analytical model and the finite element simulation. A finite element model was developed to simulate the dynamic response of the optimized auxetic NiTi structure subjected to different projectile speeds. Three stages of the impact process describing the penetration of the top plate, auxetic structure, and bottom plate have been identified. The results show that the optimized auxetic structures affect the dynamic response of the projectile by getting denser toward the impact location. This helped to improve the energy absorbed per unit mass of the NiTi auxetic structure to about two times higher than that of the solid NiTi plate and five times higher than that of the solid conventional steel plate.

## 1. Introduction

Metamaterials have attracted substantial research interest attention during the past few years as an emerging concept to develop materials with novel properties different from conventional materials, such as negative compressibility, negative Poisson’s ratio, manipulating electromagnetic radiation or sound waves, and negative elasticity. The macro/micro-structure of this type of material can be tuned to achieve desirable physical or mechanical properties, and therefore, metamaterials are also considered as designed materials. Auxetic materials, one of the metamaterials, possess a negative Poisson’s ratio, which is the ratio of the longitudinal strain to the transverse strain when the material is stretched longitudinally. The Poisson’s ratio value is positive for any conventional materials. This leads to improved shear modulus, stiffness, enhanced resistance to indentation, and improved impact energy absorption. Examples of naturally found auxetic materials include zeolites minerals, cow skin, cancellous bone, and tendons [1,2,3]. There has been a wide range of applications in using auxetic structures to develop products with desirable functionalities such as bioprostheses, shape memory foams, and running shoes. The benefits of using auxetic structures in these applications are not only because of their improved mechanical behavior but because they also hold properties such as enhanced energy absorption, shear resistance, and higher indentation resistance [4,5,6]. This has led to the advancement of auxetic metamaterials especially with the emergence of additive manufacturing (AM), which allowed designers to have the geometrical freedom to fabricate structures with a complex and controlled macro/micro-structure.

Auxetic structures can be classified into categories according to their deformation mechanism or geometry. There are three basic structures of auxetic materials: Chiral structures, re-entrant structures, and rotating rigid structures. Among them, re-entrant auxetic structures are considered the most important especially when manufactured from high-ductility material. Re-entrant structures exhibit high stability, high load-carrying capacity, and high ductility under dynamic loading [7,8,9]. 

Several research studies explored the design of auxetic materials and their behavior under high-speed impact projectiles. Schultz et al. [10] studied auxetic structures under blunt-impact projectiles similar to those used in tennis ball launchers. Four structures were produced, two with standard honeycomb hexagonal geometry, the other two with re-entrant geometry. The four structures were subjected to projectile impact with different velocities. The results showed that the auxetic structures with re-entrant unit cells absorbed more impact energy than the honeycomb ones. In addition, the re-entrant structure with a greater cell angle showed a high ballistic limit greater than 50 m/s [10]. Qi et al. [11] studied the ballistic limit and the energy absorption capability of auxetic sandwich structures. A finite element model was developed, in which a projectile with a set of velocities was used on three different panel configurations: Rectangular, hexagonal, and auxetic structures. The authors found that panels with auxetic structures have the highest ballistic limit of 190 m/s followed by hexagonal and rectangular structures, which had ballistic limits of 175 and 161 m/s, respectively. These results suggested that auxetic structures have great potential in ballistic applications compared to conventional structures [11]. Recently, a similar study was introduced by Imbalzano et al. [12]. They numerically investigated the use of several sandwich panels with cores made of an auxetic structure between two metal sheets for high-speed projectile applications. They carried out a parametric study to assess the effect of the panel configuration on the ballistic performance. The energy dissipation and deformations of the panels were assessed against conventional solid panels with the same material and equivalent mass. The study found that the auxetic panels improved the ballistic limit to about 200 m/s with a reduced deformation of 56% [12]. 

Different materials have been considered in the fabrication of auxetic structures for ballistic applications such as steel, aluminum, and titanium. Titanium alloys were found to have superior ballistic performance when compared to steel. This is because titanium alloys have low density and high strength-to-weight ratio. Gooch et al. [13] investigated the ballistic capabilities of panels made of different materials. The study found that titanium alloy has an impact energy absorption 1.3 times higher than that of steel [13]. 

Several manufacturing approaches are available to fabricate auxetic structures depending on the processed material and the intended application. Auxetic polymeric foams are typically produced using compression, heating, cooling, and relaxation of conventional foam [14]. A modified method was introduced by Webber et al., which includes a compaction process followed by two steps of sintering processes to prepare auxetic polyethylene [15]. Composite auxetic materials such as carbon fiber-reinforced polymer (CFRP) were produced using the conventional assembly method [16]. Lithographic processes such as soft lithography were also employed to prepare metal and ceramic auxetic structures [17,18], though these techniques were originally used for MEMS manufacturing [19,20,21,22,23,24,25,26,27,28,29]. 

Additive manufacturing or 3D printing is a technology to create objects layer by layer using a 3D printer according to a digital model. The technology enabling the processing of metals [30,31,32,33], ceramics [34], polymers [35], and composites [36] has been widely employed in many industries, such as biomedical [37], defense [17], aerospace [38,39], and energy [40]. The ability of AM to process a wide range of materials into objects with intricate geometries such as auxetic and cellular structures and to obtain desired mechanical properties has led to the many advancements of this technology. Four-dimensional (4D) printing is one form of 3D printing technique that has been progressed further to process materials that have the ability to respond to external stimuli, which offers products with added functionality that can be triggered without human or computer interaction. Four dimensional printing offers several advantages such as the ability to produce smart products that can change their geometries when required [17]. Choong et al. [41] used stereolithography to process a photopolymer containing a tert-butyl acrylate-co-diethylene glycol diacrylate (tBA-co-DEGDA) network with rapid curing and a shape memory effect. The authors extended the study and fabricated silica-reinforced shape memory to achieved better mechanical properties and a shape recovery ratio of 87–90% [42]. 

Powder bed fusion (PBF), which is also known as selective laser melting, is a key powder bed AM technique that can process a wide range of metal alloys with complex structures. Recently, PBF has become widely adopted in many industrial applications, offering advantages when compared to traditional manufacturing such as versatility and accuracy, as well as the ability to produce functional components. The technology demonstrated a great capability to process NiTi with high repeatability [43,44]. To improve the functionality of auxetic materials, shape memory alloys (SMA) such as NiTi can be used to combine the properties of the material to the unique behavior of the structure. NiTi SMAs exhibit motor-functionalities due to a reversible phase transformation from martensite to austenite and vice versa, which can be triggered by temperature (shape memory effect) or deformation (superelasticity). This motor-functionality makes NiTi suitable for energy-absorption, and actuating and inflatable devices as they can be actuated to their initial shape when deformed, with or without the aid of external heat [43,44,45].

This study aims to introduce a new metamaterial that combines the properties of NiTi and auxetic materials in one structure, which is processed using 4D printing. The main objectives are to design, simulate, and fabricate NiTi re-entrant structures for improved ballistic performance. Analytical and Finite Element Analysis (FEA) models were constructed and validated against the fabricated structure when subjected to compressive tests. The FEA model was then used to assess the capability of using NiTi and the auxetic structure when subject to a high-speed projectile impact.

## 2. Methodology

### 2.1. Analytical Calculations

**Figure 1:**
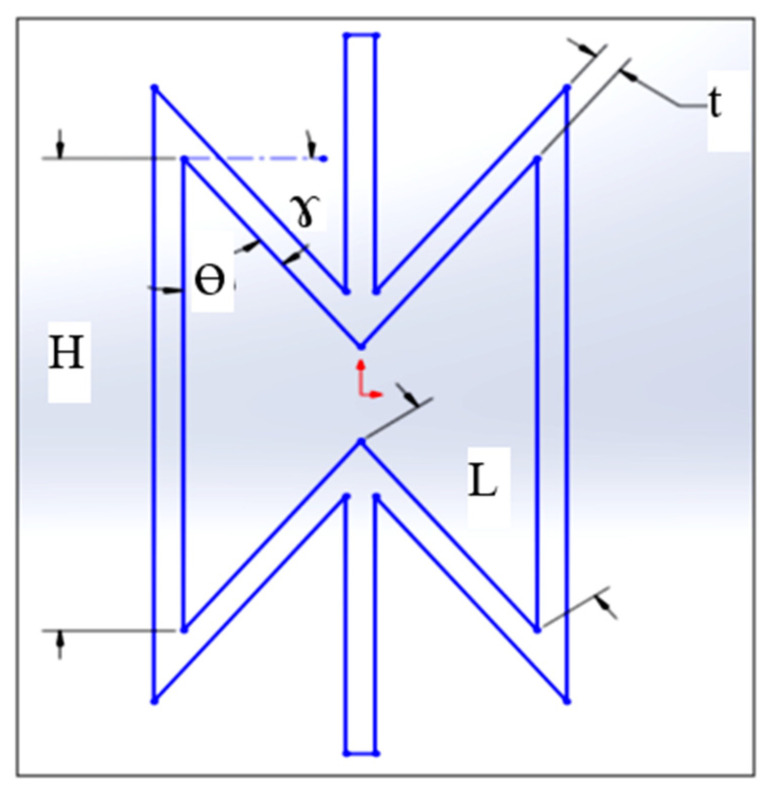
Configuration of the re-entrant unit cell.

In-plane analytical calculations were carried out on a re-entrant auxetic structure in order to obtain a high negative Poisson’s ratio before FEA modeling. Next, FEA and experimental analysis will be conducted to verify the optimization calculations. A high negative Poisson’s ratio is desirable as it enhances the associated ballistic capabilities. The geometry of the re-entrant structure is presented in Figure 1. The geometrical parameters of the re-entrant cell are thickness *t*, internal angle Ø, length *L*, and height *H* [46]. 

An analytical equation of the Poisson’s ratio of the re-entrant unit cell, introduced by Gibson and Ashby [47], was used in this study. The Poisson’s ratio equation in terms of axial and transverse deformation is given by:

(1) $$\nu =\frac{\cos^{2}\gamma }{\left(\alpha +\sin\gamma \right)\sin\gamma }\frac{1-\beta^{2}}{1+\beta^{2}\cot^{2}\gamma }$$

where *α* is the aspect ratio (*H*/*L*), *β* is the ratio (*t*/*L*), and *γ* = 90°−Ø. For auxetic structures, a geometric requirement must be added to make sure that the vertices do not overlap during deformation, which is:

(2) $$\gamma_{min}=\sin^{-1}\left(-\frac{\alpha }{2}\right)$$

In this study, the analytical model was employed to study the influence of unit cell geometrical parameters (*t*, *L*, *H*, and *γ*) on the Poisson’s ratio. Table 1 shows the geometrical parameters and the range of each one of them considering their manufacturability using PBF [10]. For each set of the cell dimensions, *α* and *β* were calculated first along with the value of *γ* before calculating the corresponding in-plane Poisson’s ratio. 

### 2.2. Modeling of Compression Testing

**Figure 2:**
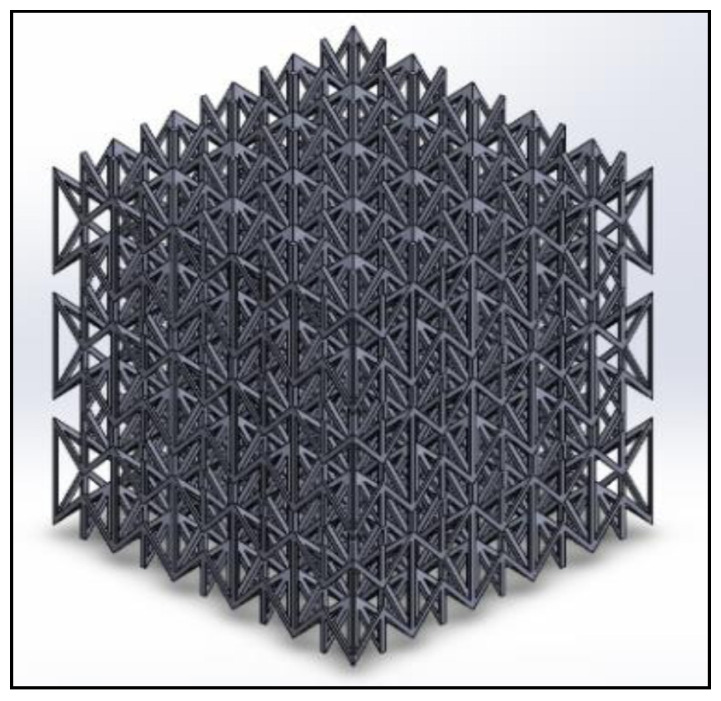
The isometric view of the optimized model.

The auxetic structure subjected to compression loading was modeled using FEA, and Poisson’s ratio was calculated and validated experimentally. The optimum geometrical parameters of the re-entrant unit cell that generated the highest in-plane negative Poisson’s ratio using the analytical model were implemented. The model was first designed using SolidWorks and the digital model was then imported into LS-DYNA for post-processing. The model consists of 5 by 5 unit cells at each face. A schematic diagram of the auxetic structure is shown in Figure 2.

The model was meshed into solid-type elements with a dimensional size of 0.52 mm. The properties of the NiTi alloy was employed using shape memory card, which provides the material behavior when undergoing a large deformation [48], see Table 2. A fixed constraint was applied at the bottom of the structure to restrict its movement and a compressive load was employed using a maximum displacement of 1 mm at the top surface of the structure.

### 2.3. 4D Printing and Characterization

**Figure 3:**
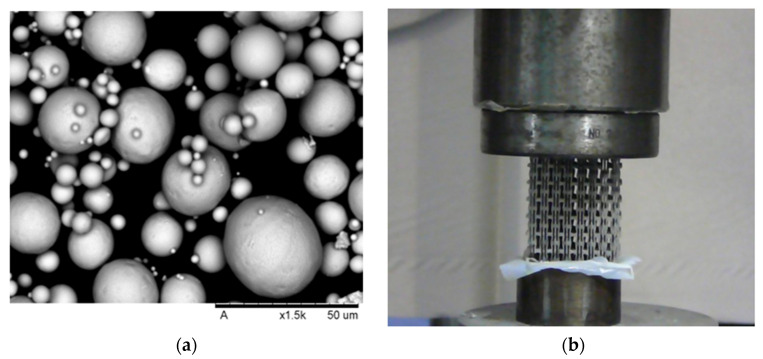
(**a**) SEM of the NiTi powder, and (**b**) the fabricated auxetic structure under compression testing.

A NiTi ingot was argon-atomized into spherical powder with d_50_ of 65 µm by (TLS Technik, Bitterfeld-Wolfen, Germany), Figure 3a. A Concept Laser M2 PBF system with an oxygen-content <0.1% was used in the fabrication process. The auxetic structure was 4D-printed using a volumetric energy density of 300 J/mm^3^, power of 70 watts, and a layer thickness of 20 µm. Differential scanning calorimetry (DSC) using a Mettler DSC 25 was carried out in an Ar atmosphere to study the phase transformation of the fabricated sample for up to a temperature of 120 °C. The fabricated samples were characterized using a Zwick Roell machine to investigate the compressive properties. Lubricated papers were used on the bottom clamping surface to reduce friction. The friction coefficient of the lubricated paper was measured and was found as 0.1. A preload of 50 N and a compression displacement speed of 1 mm/min were set. The axial and transverse displacement of the auxetic structure were recorded during loading. Figure 3b shows the samples under compression testing. 

### 2.4. Modeling of Ballistic Limit 

Finite element analysis was performed in order to investigate the performance of the optimized auxetic structures made of NiTi under the high-speed projectile impact. A comparison study with a typical solid steel alloy and NiTi plates was first performed to understand the role of the superelasticity of the NiTi on the ballistic properties. The use of a solid mild steel plate has been investigated by several researchers and, therefore, the model can be validated against experimental data from the literature. Next, the optimized NiTi auxetic model was carried out to calculate the performance against high-speed projectiles. LS-DYNA software was used to carry out the numerical simulation. Additionally, MSC Patran, ETA FEMB, and LS-PrePost were used in the pre-processing stage.

The projectile considered in the study was the 7.62 mm APM2, which is made of a core of hard steel, a brass jacket, and a lead cap. The projectile was modeled with and without the jacket and cap. It was found that modeling only the core without the other two elements of the projectile, the jacket and the cap did not significantly affect the simulation results. This is in agreement with the literature [49] as the study found that the jacket and cap can only reduce the ballistic velocity by 3–5%. As a result, only the steel core was considered in the following calculations to reduce computational time. The core dimensions are 27.6 mm in length and 6.2 mm in diameter, with a weight of 5.2 g [50]. The hard steel properties of the core are reported in Table 3. The projectile is considered as rigid, similar to reference [51]. 

The steel plate used in this study has a square geometry with dimensions of 200 mm × 200 mm and a thickness of 4.7 mm [50]. A mesh dependence study [52] was carried out based on published experimental results [50]. It was found that 3D solid elements with 1/6th the plate thickness were able to satisfactorily represent the plate behavior. However, in order to develop a more accurate model able to correctly represent the impact process with a more realistic prediction of the target damage, a finer mesh was used in the impact region. Elements near the impact zone with a 0.5 × 0.5 mm in-plane dimension and 0.52 mm thickness were used. The dimension of these elements was increased with the distance from the impact point. The plate was eliminated from the calculation when the failure strain value was reached.

The contact algorithm has to be set correctly in order to represent the real condition during the impact. The contact-eroding surface-to-surface contact algorithm was implemented as suggested in [53]. In addition, friction and heat were not considered in the modeling. The time step is one of the important tools in dynamic impact simulations. As the velocity value considered in the present analysis is very high, the time step has to be small enough to allow the correct execution of the analysis, for instance, to avoid overlap between the projectile and the plate elements during the contact. The control time step was implemented within LS-DYNA code similar to the literature [49]. 

**Figure 4:**
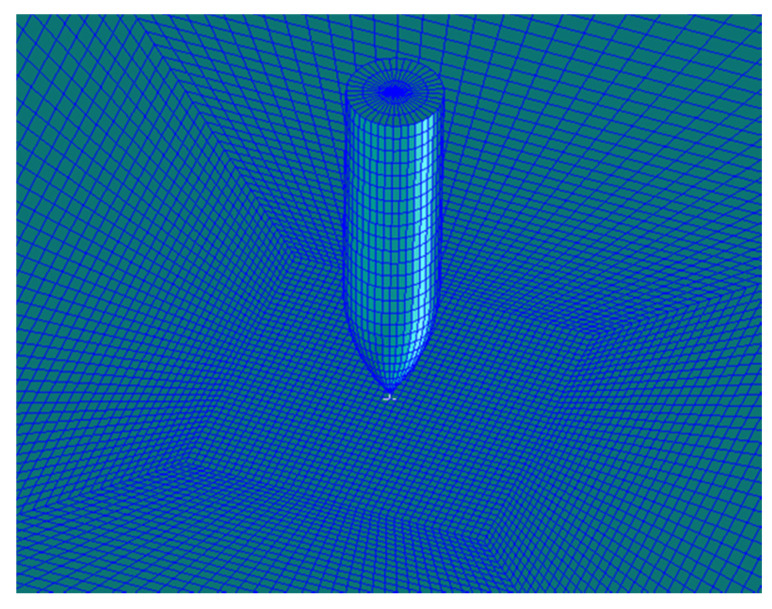
The numerical model of the projectile and solid plate.

In order to implement an appropriate hourglass control in the model, the data shown in [53] were used. A stiffness hourglass control is suggested for metal setting IHQ = 4 and considering an hourglass coefficient QH = 0.03. A comparison between the evolved internal and hourglass energy had to be performed in order to check that the model calculations are reliable. It is suggested that the model can be considered consistent if the hourglass energy is <10% of the internal energy peak [10]. The developed model of the projectile and the solid plate is shown in Figure 4. 

**Figure 5:**
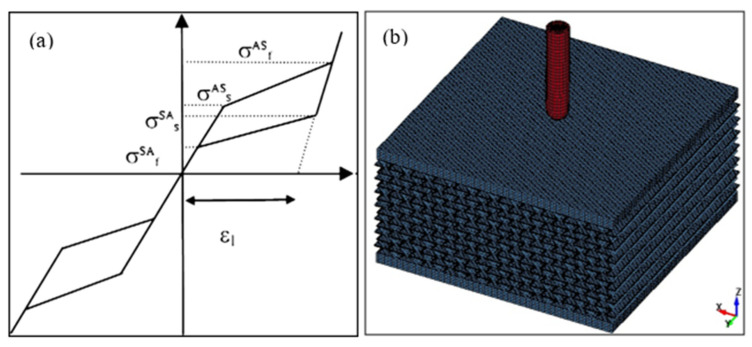

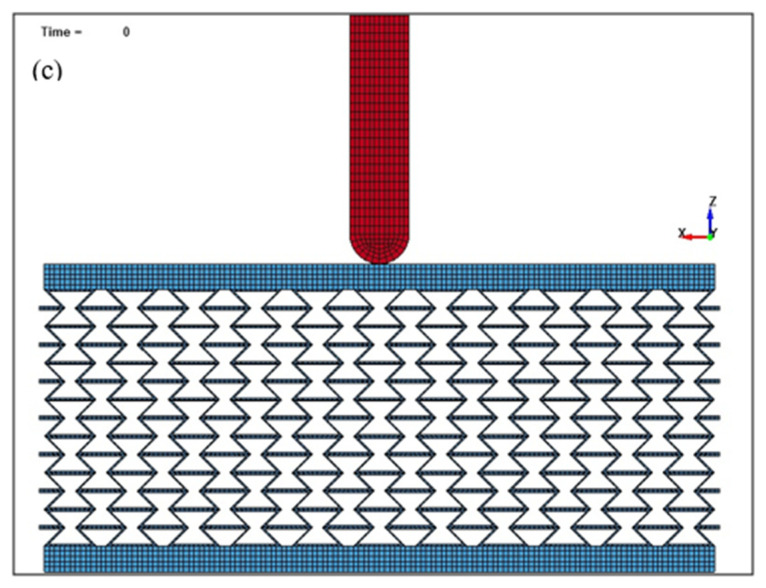
(**a**) A typical stress–strain diagram of NiTi shape memory alloy (SMA), (**b**) isometric view of the meshed model, and (**c**) front view of the meshed model.

**Figure 6:**
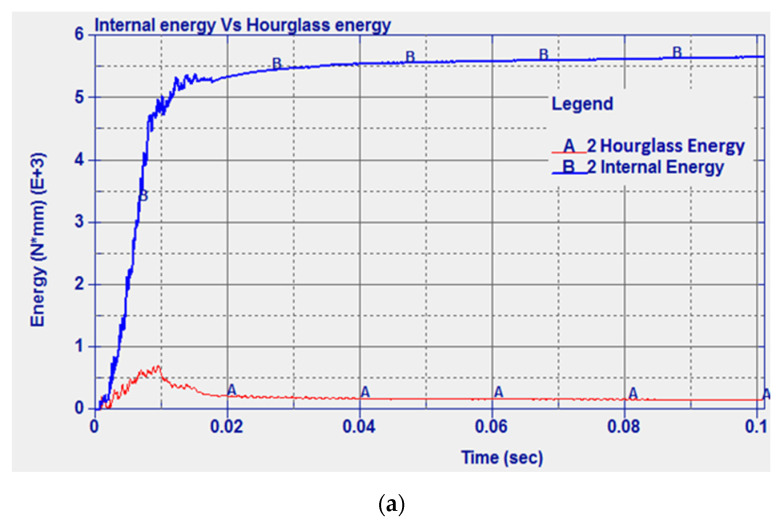

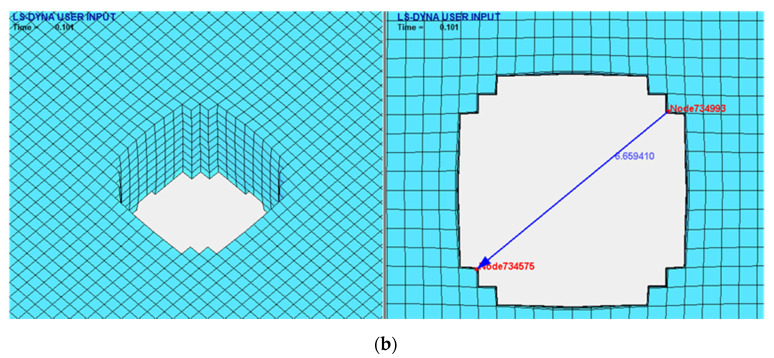
(**a**) Internal energy vs. hourglass energy, and (**b**) the 3D and 2D damage marks of the projectile penetrating the solid steel plate.

The next step is to replace the steel with the NiTi alloy. The typical behavior of shape memory alloy material is presented in Figure 5a. NiTi properties were implemented based on the data published in [54] and as shown in Table 2. The LS-DYNA material card can be used to describe the material behavior shown in Figure 6a. The condition for element failure and elimination is set (maximum principal strain at failure). Following the simulation of the solid NiTi plate subjected to a high-speed projectile, the solid NiTi plate was replaced with a re-entrant auxetic structure with two facets on the top and bottom, as shown in Figure 5b,c. The auxetic structure was 20 mm high, whereas the top and bottom facets were 2 mm in thickness. 

The auxetic structure with two facets made of NiTi was manufactured in one step using a Concept Laser M2. A striking face followed by a back face is commonly used in armoring systems. It was aimed to be thin in order to understand the role of the auxetic structure and how it behaves during penetration. A facet thickness of 2 mm was found suitable for manufacturability. We avoided using a shorter structure to allow the auxetic structure to deform and behave in an auxetic way. In our calculations, we used energy per unit mass to minimize the effect of the size of the structure. The ballistic limit was calculated using an impact speed step of 5 m/s, increasing until zero residual velocity was achieved. The speed step was refined to ± 1 m/s to improve accuracy [55].

## 3. Results and Discussion

### 3.1. Impact Simulation Using Solid Plates

For the mild steel solid plate, experimental data from the literature were used for model validation. In Figure 6a, a comparison between internal energy and hourglass energy is reported. It can be seen that the hourglass energy was much less than the internal energy peak as a result of implementing the hourglass control, which shows a reliable model. The impact velocity was 821 m/s, for which the experimental residual velocity was 758.6 m/s [49]. The numerical residual velocity was found as 789.3 m/s, which represents an error of 4.05% compared to the experimental results. The predicted results can also be compared to other numerical studies reported by Raguraman and co-authors [51] who used a rigid projectile core and shell elements for the plate. In Raguraman et al.’s study, the projectile completely left the plate after 39 ms. Similar results were obtained in the current study where 41 ms was needed for the projectile to completely penetrate the plate. The damage left by the projectile when impacting the model configuration as presented in [51] is shown in Figure 6b. The damage tends to have a near-circular geometry with 6.66 mm diameter. Similar to the experimental results, the projectile was perpendicular to the plate after the impact, and the plate tended to have major damage at the rear face. 

**Figure 7:**
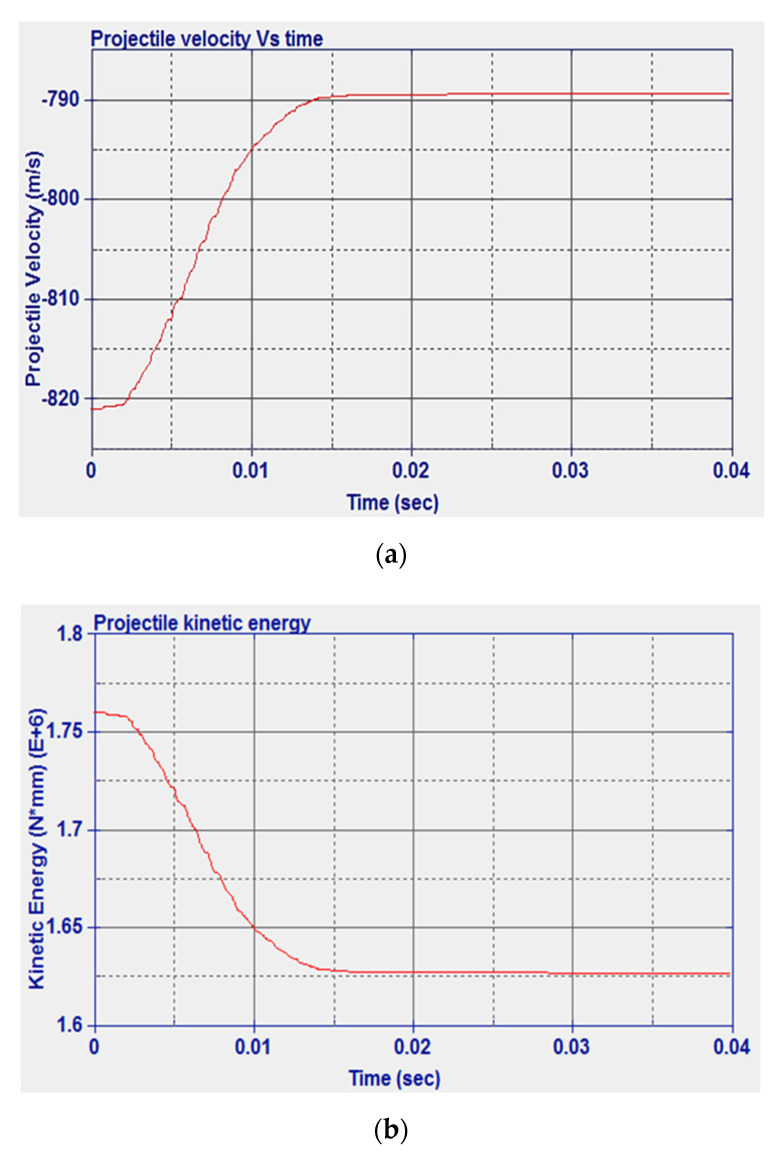
Steel solid plate: (**a**) Projectile velocity vs. time, (**b**) kinetic energy vs. time.

The history of the velocity and kinetic energy of the projectile is shown in Figure 7. From Figure 7b, it can be noted that the initial kinetic energy is 1.769 × 10^6^ N·mm and the numerical final kinetic energy is 1.635 × 10^6^ N·mm. The difference between the final and initial kinetic energies represents the numerical energy absorbed by the solid plate, and its value is 1.34 × 10^5^ N·mm. 

**Figure 8:**
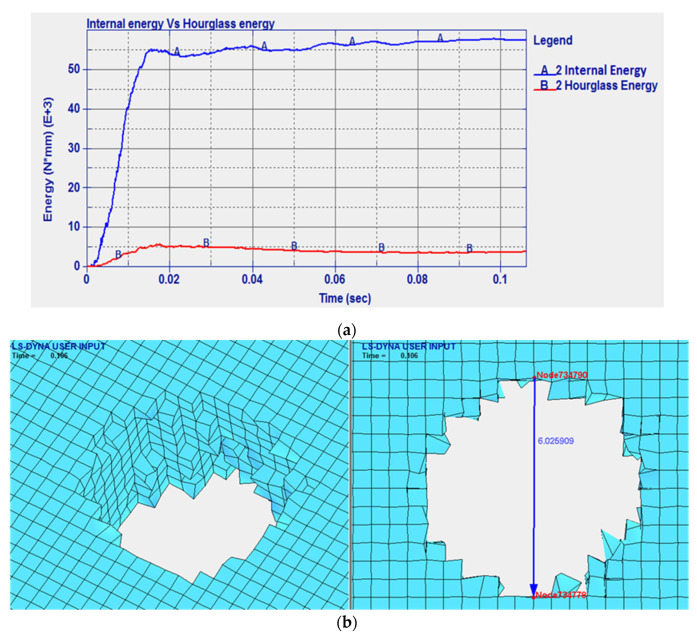
(**a**) Internal energy vs. hourglass energy, and (**b**) the 3D and 2D damage marks of the projectile penetrating the NiTi plate.

**Figure 9:**
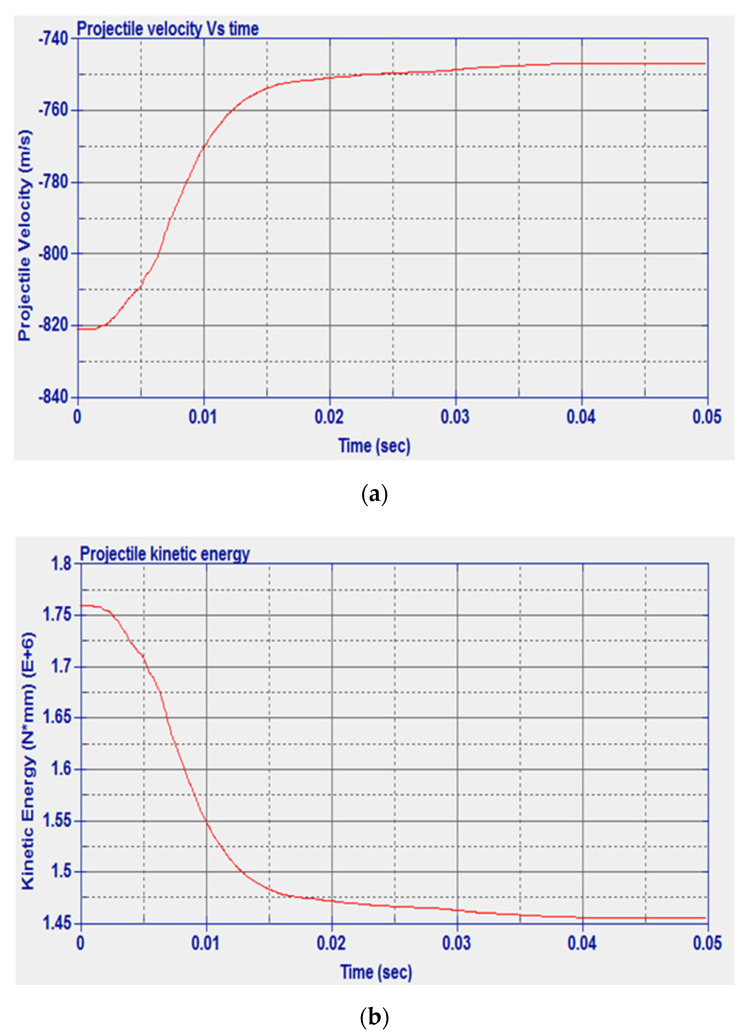
NiTi solid plate: (**a**) Projectile velocity vs. time, and (**b**) kinetic energy vs. time.

Next, the steel plate was replaced with a NiTi plate to investigate the effect of using NiTi. The impact velocity was 821 m/s, for which the numerical residual velocity was 747 m/s. As shown in Figure 8, greater internal and hourglass energy were predicted compared to those in the case of steel. The hourglass energy was still less than 10% of the internal energy peak owing to the implemented hourglass control, see Figure 8a. The damage left by the projectile was smaller than that in the case of steel with a 6.02 mm diameter compared to the 6.66 mm diameter when the steel plate was used as shown in Figure 8b. Figure 9 presents the variation in the velocity and the kinetic energy of the projectile over time. As shown, the initial projectile kinetic energy is 1.769 × 10^6^ N·mm. The difference between the initial numerical kinetic energy and the final kinetic energy represents the numerical energy absorbed by the plate, which is 3.06 × 10^5^ N·mm.

Table 4 shows a comparison between the ballistic performance of both the steel and NiTi. It is clear that the NiTi plate experienced better ballistic behavior when hit by a high-speed projectile. The residual velocity of the projectile and the damage in the target were lower than those in the steel plate. As a result, the absorbed energy by the plate was higher. An important conclusion is that the absorbed energy by a unit mass of NiTi is about 2.7 times that in steel.

### 3.2. Optimization of Poisson’s Ratio and Validation 

**Figure 10:**
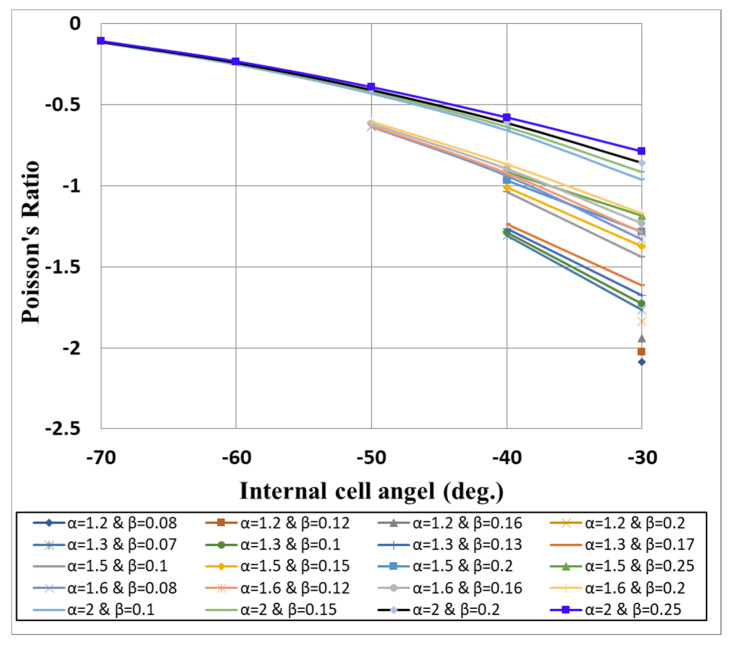
In-plane Poisson’s ratio calculations as a function of cell dimensions.

Figure 10 shows the analytically calculated Poisson’s ratio for different unit cell parameters. As shown, the values of Poisson’s ratio were obtained (from Equation (1)) and plotted against the cell angle for different aspect ratios α and densities β. The curves were interrupted for cell angles lower than the critical angle introduced in Equation (2), γ_min_. It can be noted that a negative cell angle resulted in a negative Poisson’s ratio. In addition, small and negative cell angles combined with low aspect ratio would result in increasing the negative Poisson’s ratio. Furthermore, the Poisson’s ratio had direct relationships with both α and β. By increasing the aspect ratio from 1.2 to 2 (at a cell angle of −30° and a relative density of 0.2), the Poisson’s ratio increased from −1.84 to −0.86. On the other hand, increasing the relative density from 0.1 to 0.25 (at a cell angle of −40° and an aspect ratio of 1.5) increased the Poisson’s ratio from −1.04 to −0.91. This means that not only the cell angle but also the other geometric cell parameters can dominate the values of the re-entrant structure Poisson’s ratio. Finally, it should be emphasized that as the values of the relative density in this work were relatively small (β ≤ 0.25), the deflection of the walls can be neglected [46]. From the results of the analytical model, the cell parameters that yielded the minimum Poisson’s ratio were found to be α = 1.2, β = 0.08, and γ = −30°. The corresponding Poisson’s ratio was calculated to be −2.089. These values of cell parameters were used further during the FE modeling and experimental study. 

**Figure 11:**
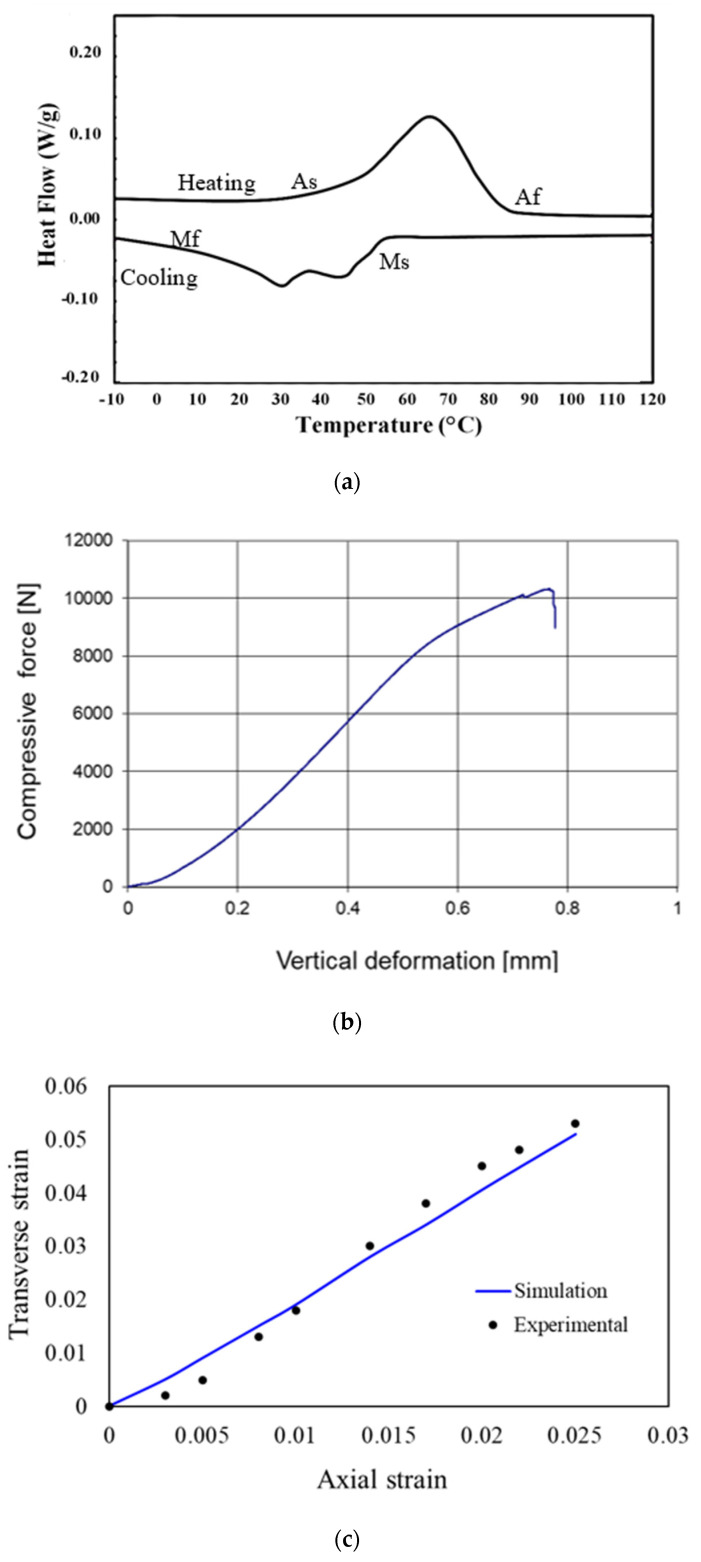
(**a**) DSC curve of the fabricated sample, (**b**) force−displacement curve of one of the fabricated samples using compression testing, and (**c**) experimental and simulated compressive transverse strain versus compressive axial strain for the NiTi auxetic structure.

Figure 11 shows the DCS, force−displacement, and experimental and simulated transverse strain against the axial strain for the re-entrant auxetic structure. The DSC curve of the fabricated sample demonstrates the phase transformation from austenite and martensitic during heating and cooling. Overall, the results showed that the sample preserved the shape memory characteristics of NiTi, Figure 11a. The maximum compressive force for the samples was 10 kN, Figure 11b. Following compression, the deformed structure was heated to 100 °C, which led to the restoration of the original (undeformed) geometry. Figure 11c shows a good agreement between the experimental and simulation results. The calculated Poisson’s ratio was obtained by determining the slope of the simulation and experimental lines. The Poisson’s ratio of simulation results is −2.04, whereas it is −1.98 from the experimental results. The results show an acceptable modeling error of <5% between the experimental and simulation values. In addition, the simulation and the experimentally obtained data are close to the analytically obtained in-plane Poisson’s ratio of −2.089. The agreement between the analytical, simulation, and literature results enabled the progression of the ballistic modeling. 

### 3.3. Impact Simulation Using NiTi Auxetic Structure

In this section, the re-entrant auxetic structure with the optimum parameters was employed with solid facets on the top and bottom, as represented in Figure 5c. The auxetic structure is 20 mm high plus top and bottom plates each with 2 mm thickness. Different impact velocities (Vi), ranging from 875 to 900 m/s, were used, and the energy absorption and the residual velocity during the impact were calculated. The evolution of the projectile velocity at several impact velocities is depicted as a function of time in the following set of figures. Figures of two projectile velocities of 875 to 900 m/s are presented to avoid repeatability. The total time was estimated to have a maximum of 0.16, which can be defined as the time when the projectile touches the top plate and the time at which the projectile pierces the structure. 

**Figure 12:**
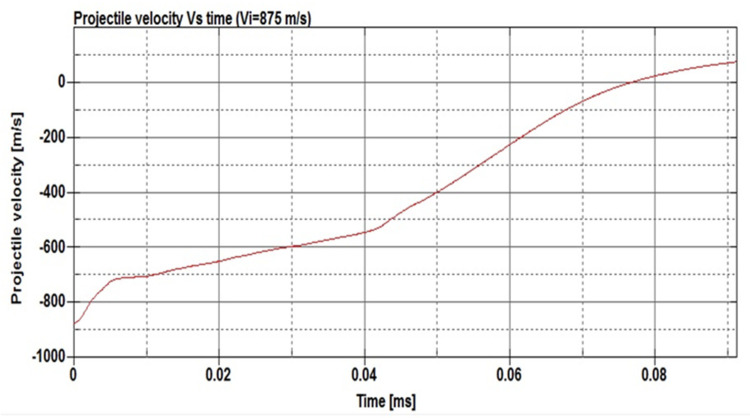
Projectile velocity vs. time for the NiTi auxetic structure at a projectile speed of 875 mm/s.

**Figure 13:**
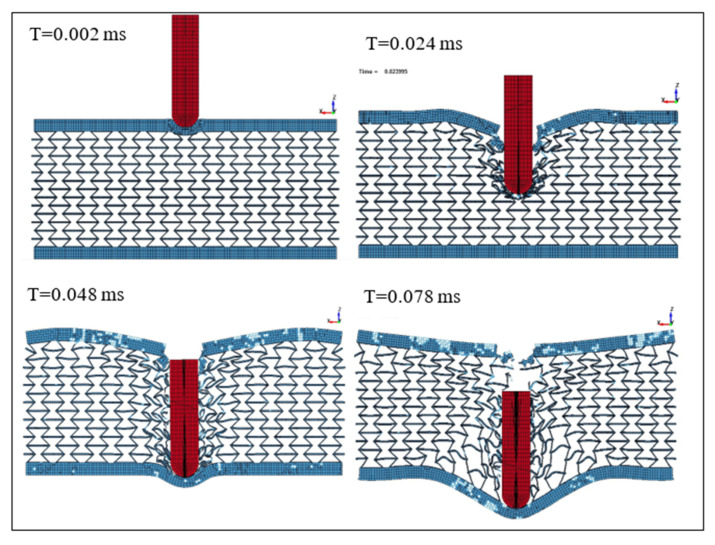
Structure deformation at different timings, Vi = 875 m/s.

**Figure 14:**
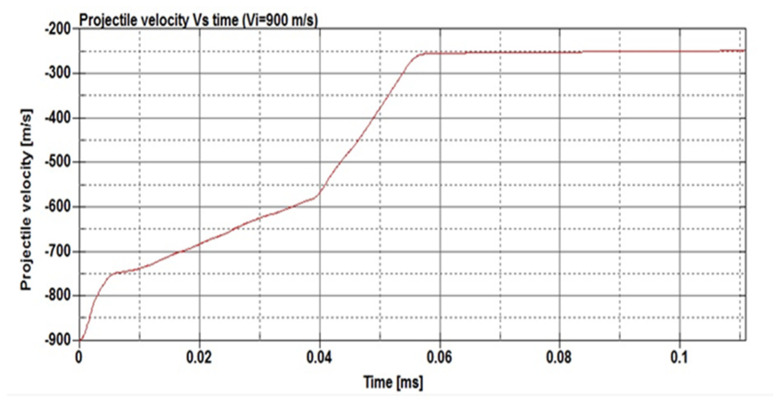
Projectile velocity vs. time for the NiTi auxetic structure at a projectile speed of 900 mm/s.

**Figure 15:**
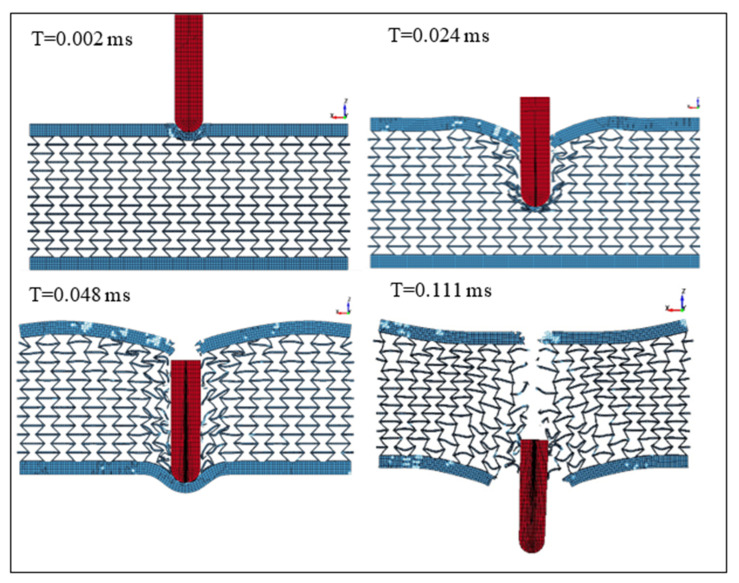
Structure deformation at different timings, Vi = 900 m/s.

Figure 12 presents the changes in the projectile velocity as the time of the impact increases. The energy absorbed per unit mass was determined to be 495 N·mm/g. On the other hand, Figure 13 shows the deformation of the SMA plate during the impact at different time steps from 0.002 to 0.078 ms. The simulation was repeated at a projectile speed of 900 m/s. The projectile velocity and the energy values in the plate during the impact are given in Figure 14. In this case, the energy absorbed per unit mass was 521 N.mm/g. Furthermore, the deformation of the SMA auxetic structure during the impact at different time steps from 0.002 to 0.111 ms is shown in Figure 15.

Figure 12, Figure 13, Figure 14 and Figure 15 show that three different stages can be identified during the penetration process of the projectile into the auxetic structure: The top plate, auxetic structure, and bottom plate. In stage 1, at the beginning of the impact event, the top plate caused a sudden drop in the projectile velocity, so the projectile approached the auxetic structure at a velocity of about 420 m/s. In stage 2, the auxetic structure became denser in the region around the projectile and, hence, the projectile velocity decreased as the projectile penetrated through the auxetic structure. It can be concluded that the auxetic structure tends to get closer and denser trying to resist the penetration of the projectile, which is very important to absorb more energy from the projectile impact. In stage 3, an additional drop in the projectile velocity was caused by the bottom plate. It can be noted that the developed internal energy in the structure increases when the projectile impact speed increases. The ballistic limit of the structure, which is the velocity at which the projectile cannot penetrate through the whole armor layer, can be calculated. In other words, the residual velocity of projectile becomes zero when the impact velocity of the projectile is lower than the ballistic limit. In this case, at a projectile impact speed of 875 m/s, the projectile velocity reached zero m/s at the time of 0.078 ms, and the projectile could not pierce into the armor structure. The calculated energy absorbed by unit mass, in this case, is 495 N·mm/g compared to 91.28 and 252.35 N·mm/g for steel and NiTi solid plates, respectively. Finally, at higher speeds, the projectile could pierce the structure and leave with a residual velocity.

## 4. Conclusions

This paper introduced a metamaterial that combines the shape memory/superelasticity and negative Poisson’s ratio properties, and was proved to have higher ballistic performance when compared to conventional steel armors. An analytical model was first used to optimize the geometrical parameters of the re-entrant auxetic structure. It was found that the re-entrant auxetic structure with a cell angle of −30° produced the highest Poisson’s of −2.089. The 4D printing process using PBF was used to fabricate the optimized auxetic structure using gas-atomized NiTi powder. The obtained Poisson’s ratio of the fabricated auxetic structure was found in agreement with both the analytical model and the finite element simulation. A finite element model was developed of a hard steel core projectile and both NiTi and steel solid plates to understand the effect of using NiTi when compared to the conventional steel material. The results show that NiTi proved to have a better performance than steel. The impact processes were also simulated to the optimized auxetic NiTi structure at different projectile speeds using a nonlinear FE code. Three stages of the impact process describing the penetration of the top plate, auxetic structure, and bottom plate were clearly identified. The results also show that the optimized auxetic structures affect the dynamic response of the projectile by getting denser toward the impact location. This helped to improve the energy absorbed by unit mass from 91.28 and 252.35 N·mm/g in the case of solid steel and solid NiTi, respectively, to 495 N·mm/g for the optimized NiTi auxetic structure. Future studies will include the implementation of the developed auxetic structure into existing armor designs. The ballistic performance will be experimentally investigated with projectiles at different speeds, and the compression characterization will be studied at different strain rates to capture the implications and limitations of the developed metamaterial.

## Figures and Tables

**Table 1:** Re-entrant unit cell geometrical parameters and their corresponding ranges.

Parameter	Min. Value	Max. Value
*H* (mm)	2	4
*L* (mm)	2	3
*t* (mm)	0.2	0.5
*γ* (degree)	−70	−30

**Table 2:** Properties of NiTi shape memory alloy [48].

Melting Point (°C)	1300
Young modulus (GPa)	29.3
Density (g/cm^3^)	6.45
Poisson’s ratio	0.33
$\sigma_{F}^{AS}\;\left(\mathrm{MPa}\right)$	896
$\sigma_{S}^{AS}\;\left(\mathrm{MPa}\right)$	790
$\sigma_{S}^{AS}\;(\mathrm{MPa})$	600
$\sigma_{F}^{AS}\;\left(\mathrm{MPa}\right)$	450
^a^ ε_1_	0.078
^b^ ε_2_	0.08
^c^ α	0
^d^ ΔE	0
^e^ ε_pmax_	0.175

^a^ ε_1_ is the strain at the end of martensite-to-austenite transformation; ^b^ ε_2_ is the strain at the end of austenite-to-austenite transformation; ^c^ α is a parameter measuring the difference between material responses in tension and compression (set α = 0 for no difference); ^d^ ΔE is Young’s modulus for the martensite phase if different from the modulus for the austenite. Set to zero if equal to the austenite modulus; ^e^ ε_pmax_ is the maximum principal strain at failure.

**Table 3:** Properties of the core and the solid plate, [49].

Material	Density [kg/m^3^]	Young’s Modulus [GPa]	Poisson’s Ratio	Yield Stress [MPa]	E _tan_ [GPa]
Hard steel	7850	203.4	0.30		
Mild steel	7810	205.4	0.30	205	80

**Table 4:** Comparison between steel and NiTi shape memory alloy plate.

Parameter	Steel	NiTi
Weight of the target (g)	1468	1212.6
Thickness (mm)	4.7	4.7
The residual velocities (mm/sec)	758	746
The energy absorbed (N·mm)	1.34 × 10^5^	3.06 × 10^5^
Energy absorbed by unit mass (N·mm/g)	91.28	252.35
Damage (mm)	6.66	6.02

## References

[1] Williams J.L., Lewis J.L. (1982). Properties and an anisotropic model of cancellous bone from the proximal tibial epiphysis. J. Biomech. Eng..

[2] Gatt R., Wood M.V., Gatt A., Zarb F., Formosa C., Azzopardi K.M., Casha A., Agius T.P., Schembri-Wismayer P., Attard L. (2015). Negative Poisson’s ratios in tendons: An unexpected mechanical response. Acta Biomater..

[3] Kimizuka H., Kaburaki H., Kogure Y. (2000). Mechanism for negative poisson ratios over the alpha- beta transition of cristobalite, SiO_2_: A molecular-dynamics study. Phys. Rev. Lett..

[4] Sanami M., Ravirala N., Alderson K., Alderson A. (2014). Auxetic materials for sports applications. Procedia Eng..

[5] Santo L. (2016). Shape memory polymer foams. Prog. Aerosp. Sci..

[6] Scarpa F. (2008). Auxetic materials for bioprostheses [In the Spotlight]. IEEE Signal Process. Mag..

[7] Boldrin L., Hummel S., Scarpa F., di Maio D., Lira C., Ruzzene M., Remillat C.D.L., Lim T.C., Rajasekaran R., Patsias S. (2016). Dynamic behaviour of auxetic gradient composite hexagonal honeycombs. Compos. Struct..

[8] Hajmohammad M.H., Nouri A.H., Zarei M.S., Kolahchi R. (2019). A new numerical approach and visco-refined zigzag theory for blast analysis of auxetic honeycomb plates integrated by multiphase nanocomposite facesheets in hygrothermal environment. Eng. Comput..

[9] Imbalzano G., Tran P., Ngo T.D., Lee P.V.S. (2016). A numerical study of auxetic composite panels under blast loadings. Compos. Struct..

[10] Schultz J. (2011). Modeling and Finite Element Analysis Methods for the Dynamic Crushing of Honeycomb Cellular Meso-Structures. Master’s Thesis.

[11] Qi C., Yang S., Wang D., Yang L.J. (2013). Ballistic resistance of honeycomb sandwich panels under in-plane high-velocity impact. Sci. World J..

[12] Imbalzano G., Tran P., Ngo T.D., Lee P.V.S. (2017). Three-dimensional modelling of auxetic sandwich panels for localised impact resistance. J. Sandw. Struct. Mater..

[13] Gooch W.A. Potential applications of titanium alloys in armor aystems. Proceedings of the Titanium 2011 International Titanium Association.

[14] Chan N., Evans K.E. (1997). Fabrication methods for auxetic foams. J. Mater. Sci..

[15] Webber R.S., Alderson K.L., Evans K.E. (2008). A novel fabrication route for auxetic polyethylene, part 2: Mechanical properties. Polym. Eng. Sci..

[16] Yao Y., Luo Y., Xu Y., Wang B., Li J., Deng H., Lu H. (2018). Fabrication and characterization of auxetic shape memory composite foams. Compos. Part B Eng..

[17] Lantada A.D., Romero A.d., Schwentenwein M., Jellinek C., Homa J. (2016). Lithography-based ceramic manufacture (LCM) of auxetic structures: Present capabilities and challenges. Smart Mater. Struct..

[18] Xu B., Arias F., Brittain S.T., Zhao X.M., Grzybowski B., Torquato S., Whitesides G.M. (1999). Making negative Poisson’s ratio microstructures by soft lithography. Adv. Mater..

[19] Hassanin H., Jiang K. (2009). Alumina composite suspension preparation for softlithography microfabrication. Microelectron. Eng..

[20] Hassanin H., Jiang K. (2013). Fabrication and characterization of stabilised zirconia micro parts via slip casting and soft moulding. Scr. Mater..

[21] Hassanin H., Jiang K. (2009). Fabrication of Al_2_O_3_/SiC Composite Microcomponents using Non-aqueous Suspension. Adv. Eng. Mater..

[22] Hassanin H., Jiang K. (2011). Multiple replication of thick PDMS micropatterns using surfactants as release agents. Microelectron. Eng..

[23] Hassanin H., Jiang K. (2013). Net shape manufacturing of ceramic micro parts with tailored graded layers. J. Micromech. Microeng..

[24] Zhu Z., Hassanin H., Jiang K. (2010). A soft moulding process for manufacture of net-shape ceramic microcomponents. Int. J. Adv. Manuf. Technol..

[25] El-Sayed M.A., Hassanin H., Essa K. (2016). Bifilm defects and porosity in Al cast alloys. Int. J. Adv. Manuf. Technol..

[26] Hassanin H., Jiang K. (2010). Functionally graded microceramic components. Microelectron. Eng..

[27] Essa K., Modica F., Imbaby M., El-Sayed M.A., ElShaer A., Jiang K., Hassanin H. (2017). Manufacturing of metallic micro-components using hybrid soft lithography and micro-electrical discharge machining. Int. J. Adv. Manuf. Technol..

[28] Hassanin H., Jiang K. (2010). Optimized process for the fabrication of zirconia micro parts. Microelectron. Eng..

[29] El-Sayed M.A., Hassanin H., Essa K. (2016). Effect of casting practice on the reliability of Al cast alloys. Int. J. Cast Met. Res..

[30] Qiu C., Adkins N.J.E., Hassanin H., Attallah M.M., Essa K. (2015). In-situ shelling via selective laser melting: Modelling and microstructural characterization. Mater. Des..

[31] Hassanin H., Alkendi Y., Elsayed M., Essa K., Zweiri Y. (2020). Controlling the properties of additively manufactured cellular structures using machine learning approaches. Adv. Eng. Mater..

[32] Hassanin H., Essa K., Qiu C., Ali M.A., Nicholas J.E.A., Moataz M.A. (2017). Net-shape manufacturing using hybrid selective laser melting/hot isostatic pressing. Rapid Prototyp. J..

[33] Essa K., Khan R., Hassanin H., Attallah M.M., Reed R. (2016). An iterative approach of hot isostatic pressing tooling design for net-shape IN718 superalloy parts. Int. J. Adv. Manuf. Technol..

[34] Essa K., Hassanin H., Attallah M.M., Adkins N.J., Musker A.J., Roberts G.T., Tenev N., Smith M. (2017). Development and testing of an additively manufactured monolithic catalyst bed for HTP thruster applications. Appl. Catal. A Gen..

[35] Klippstein H., Hassanin H., De Cerio Sanchez A.D., Zweiri Y., Seneviratne L. (2018). Additive manufacturing of porous structures for unmanned aerial vehicles applications. Adv. Eng. Mater..

[36] Li Y., Feng Z., Huang L., Essa K., Bilotti E., Zhang H., Peijs T., Hao L. (2019). Additive manufacturing high performance graphene-based composites: A review. Compos. Part A Appl. Sci. Manuf..

[37] Hassanin H., Finet L., Cox S.C., Jamshidi P., Grover L.M., Shepherd D.E.T., Addison O., Attallah M.M. (2018). Tailoring selective laser melting process for titanium drug-delivering implants with releasing micro-channels. Addit. Manuf..

[38] Klippstein H., De Cerio Sanchez A.D., Hassanin H., Zweiri Y., Seneviratne L. (2018). Fused deposition modeling for unmanned aerial vehicles (uavs): A review. Adv. Eng. Mater..

[39] Galatas A., Hassanin H., Zweiri Y., Seneviratne L. (2018). Additive manufactured sandwich composite/ABS parts for unmanned aerial vehicle applications. Polymers.

[40] Sabouri A., Yetisen A.K., Sadigzade R., Hassanin H., Essa K., Butt H. (2017). Three-Dimensional Microstructured Lattices for Oil Sensing. Energy Fuels.

[41] Choong Y.Y.C., Maleksaeedi S., Eng H., Yu S., Wei J., Su P.C. (2020). High speed 4D printing of shape memory polymers with nanosilica. Appl. Mater. Today.

[42] Choong Y.Y.C., Maleksaeedi S., Eng H., Wei J., Su P.C. (2017). 4D printing of high performance shape memory polymer using stereolithography. Mater. Des..

[43] Li S., Hassanin H., Attallah M.M., Adkins N.J.E., Essa K. (2016). The development of TiNi-based negative Poisson’s ratio structure using selective laser melting. Acta Mater..

[44] Tan C., Li S., Essa K., Jamshidi P., Zhou K., Ma W., Attallah M.M. (2019). Laser powder bed fusion of Ti-rich TiNi lattice structures: Process optimisation, geometrical integrity, and phase transformations. Int. J. Mach. Tools Manuf..

[45] Hassanin H., Kinnni A., ElShaer A., Polycarpou E., Elsayed M., Essa K. (2017). Stainless steel with tailored porosity using canister-free hot isostatic pressing for improved osseointegration implants. J. Mater. Chem. B.

[46] Scarpa F., Panayiotou P., Tomlinson G. (2000). Numerical and experimental uniaxial loading on in-plane auxetic honeycombs. J. Strain Anal. Eng. Des..

[47] Gibson L.J., Ashby M.F. (1982). The mechanics of three-dimensional cellular materials. Proc. R. Soc. Lond. A Math. Phys. Eng. Sci..

[48] Nagraj R.G., Venkatesha C., Jain R. (2012). Numerical simulation of soft-body impact on Shape Memory Alloys (SMA). Int. J. Sci. Eng. Res..

[49] Kılıç N., Ekici B. (2013). Ballistic resistance of high hardness armor steels against 7.62 mm armor piercing ammunition. Mater. Des..

[50] Gupta N., Madhu V. (1997). An experimental study of normal and oblique impact of hard-core projectile on single and layered plates. Int. J. Impact Eng..

[51] Raguraman M., Deb A., Gupta N. (2007). A numerical study of projectile impact on mild steel armour plates. Curr. Sci..

[52] Raguraman M., Deb A. Accurate prediction of projectile residual velocity for impact on single and multi-layered steel and aluminum plates. Proceedings of the 9th International LS-DYNA Users Conference.

[53] Bala S., Day J. (2012). General Guidelines for Crash Analysis in LS-DYNA.

[54] Meo M., Marulo F., Guida M., Russo S. (2013). Shape memory alloy hybrid composites for improved impact properties for aeronautical applications. Compos. Struct..

[55] Serjouei A., Chi R., Zhang Z., Sridhar I. (2015). Experimental validation of BLV model on bi-layer ceramic-metal armor. Int. J. Impact Eng..

